# Development of an antibody fragment that stabilizes GPCR/G-protein complexes

**DOI:** 10.1038/s41467-018-06002-w

**Published:** 2018-09-13

**Authors:** Shoji Maeda, Antoine Koehl, Hugues Matile, Hongli Hu, Daniel Hilger, Gebhard F. X. Schertler, Aashish Manglik, Georgios Skiniotis, Roger J. P. Dawson, Brian K. Kobilka

**Affiliations:** 1 Department of Molecular and Cellular Physiology, Stanford University School of Medicine, 279 Campus Drive, Stanford, CA 94305 USA; 2 Department of Structural Biology, Stanford University School of Medicine, 279 Campus Drive, Stanford, CA 94305 USA; 3 Roche Pharma Research and Early Development, Therapeutic Modalities, Roche Innovation Center Basel, F.Hoffmann-La Roche Ltd, Grenzacherstrasse 124, 4070 Basel, Switzerland; 4 Laboratory of Biomolecular Research, Paul Scherrer Institute, 5232 Villigen, Switzerland; 5 Department of Pharmaceutical Chemistry, University of California San Francisco, 1700 4th Street, San Francisco, CA 94143 USA; 6 Department of Anesthesia and Perioperative Care, University of California San Francisco, 1700 4th Street, San Francisco, CA 94143 USA

## Abstract

Single-particle cryo-electron microscopy (cryo-EM) has recently enabled high-resolution structure determination of numerous biological macromolecular complexes. Despite this progress, the application of high-resolution cryo-EM to G protein coupled receptors (GPCRs) in complex with heterotrimeric G proteins remains challenging, owning to both the relative small size and the limited stability of these assemblies. Here we describe the development of antibody fragments that bind and stabilize GPCR-G protein complexes for the application of high-resolution cryo-EM. One antibody in particular, mAb16, stabilizes GPCR/G-protein complexes by recognizing an interface between Gα and Gβγ subunits in the heterotrimer, and confers resistance to GTPγS-triggered dissociation. The unique recognition mode of this antibody makes it possible to transfer its binding and stabilizing effect to other G-protein subtypes through minimal protein engineering. This antibody fragment is thus a broadly applicable tool for structural studies of GPCR/G-protein complexes.

## Abstract (web summary)

The determination of high resolution structures of G protein coupled receptors (GPCRs) in complex with heterotrimeric G proteins is challenging. Here authors develop an antibody fragment, mAB16, which stabilizes GPCR/G-protein complexes and facilitates the application of high resolution cryo-EM.

## Introduction

G-protein coupled receptors (GPCRs) make up the largest receptor family in the human genome, comprising around 800 members. GPCRs are expressed ubiquitously and play essential roles of signal transduction in response to a wide variety of extracellular stimuli such as photons, ions, neurotransmitters, hormones and proteins. Given their numerous physiological roles, GPCRs are implicated in numerous diseases and ~30% of marketed drugs are targeting this receptor family^1^. Recent advances in GPCR crystallography have led to high-resolution structures of G-protein^2^ and arrestin^3^ complexes, which have enhanced our understanding of the structural details underlying ligand binding and signal transduction at the atomic level. The first crystal structure of a GPCR/G-protein complex was that of the β_2_ adrenergic receptor in complex with stimulatory G-protein, G_s_ (β_2_AR/G_s_)^2^. This was later followed by the crystal structure of A_2A_ adenosine receptor in complex with miniG_s_ (A_2A_R/miniG_s_) in which a highly engineered Gα_s_ that consists of only the Gα ras-like domain was used in place of the full heterotrimer^2,4^. The fact that such drastic protein engineering is needed to obtain diffraction quality crystals reflects the difficulty inherent in GPCR–G-protein complex crystallography. Despite the technological advancement, crystallographic studies of these complexes remains extremely difficult. More recently, single-particle cryo-electron microscopy (cryo-EM) has emerged as an alternative technique with the ability to provide near-atomic resolution maps, as demonstrated for two class B GPCRs^3,5,6^ both in complex with G_s_: the glucagon-like peptide1 receptor/G_s_ (Glp-1R/G_s_)^6,7^ as well as the calcitonin receptor/G_s_ (CTR/G_s_)^5^. These studies have highlighted the possibility of employing cryo-EM to obtain the structures of GPCR-G protein complexes. Compared to class A GPCRs, class B receptors include a structured extracellular domain that may aid in particle alignment. Furthermore, for Gs proteins, Nb35^2^ stabilizes these complexes against GTPγS by stabilizing an interface between the Ras-like domain of the Gα_s_ subunit and the Gβ subunit. These factors make class B GPCR/Gs protein complexes more tractable targets for cryo-EM compared to class A or other G-protein subtype complexes.

Apart from the GPCR/G_s_ complex, the only structure available at high-resolution has been limited to the MetaII rhodopsin/Gα_Ct_ where the last 11-amino-acid fragment of G_transducin_ was co-crystalized with the activated rhodopsin^8^. Although in silico analyses using this complex have provided insights into the conformational changes that allow G_i_ coupling as well as general principles for G protein coupling specificity^9,10^, experimental structures of other G-protein complexes are invaluable to understand how receptors selectively engage one G-protein subtype over others. G-protein mimetic nanobodies have been used as a surrogate to capture the active conformation of a receptor^11–14^, but it may require an extensive effort to find such nanobodies and the trapped conformation may not necessarily represent the G-protein engaged state.

Here we describe the development of an antibody, termed mAb16, that recognizes the heterotrimeric G_i/o_ type G protein and enhances the stability of GPCR-Gi/o complexes, while simultaneously adding an asymmetric feature that may aid with cryo-EM particle projection alignment. As antibodies typically bind to their targets in a rigid manner, such an antibody would be expected to enable structure determination of GPCR/G-protein complexes by cryo-EM. mAb16 recognizes a unique epitope, binding at the interface between the α and β subunits of heterotrimeric G_i_. While the antibody confers extra stability to GPCR/G_i/o_ complex as well as increased resistance to GTPγS-triggered dissociation of the complex in a manner similar to Nb35 for G_s_, mAb16 and Nb35 bind to completely different epitopes. We have recently succeeded in obtaining a near-atomic resolution map of the mu-opioid receptor (μOR)/G_i_ complex using this antibody fragment^15^. Although this antibody is specific against G_i/o_-family G-proteins, its ability to bind and stabilize the heterotrimer can be transferred to other G-protein subtypes through a simple protein engineering strategy.

## Results

### Selection of monoclonal antibodies

**Fig. 1:**
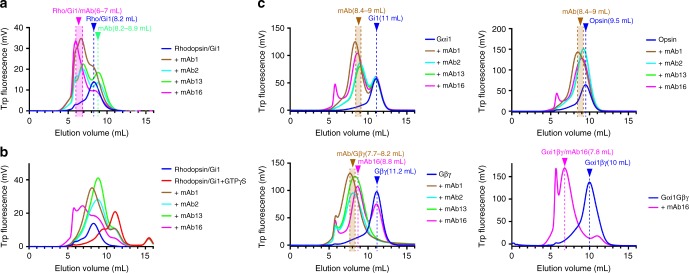
Isolation of mAb16 and its binding profile to each component. **a** Analytical SEC of rhodopsin/G_i1_ with each antibody. Rhodopsin/G_i1_ runs at 8.2 mL and each mAb alone runs 8.4–9 mL (**c**). Rhodopsin/G_i1_ bound to mAb makes higher molecular weight product and migrates at the elution volume of 6–7 mL depending on the mAb. **b** Analytical SEC of rhodopsin/G_i1_ with each antibody following to GTPγS treatment. Intact complex remains at 6–7 mL only in the mAb16 condition. **c** Analytical SEC of individual component of rhodopsin/G_i1_ or heterotrimeric G_i1_ with each antibody. Top left: Binding experiment with Gαi1 subunit and each mAb. The peak of Gαi1 at 11 mL stays intact indicating there is no binding with each mAb. Top right: Binding experiment with opsin. Both mAb peaks and Opsin peak (at 9.5 mL) stays intact. Bottom left: Binding experiment with Gβγ subunit. The peak of Gβγ at 11.2 mL disappears upon incubating with mAbs except mAb16 and each mAb peak shifts towards left compared to the ones with Gαi1 or opsin indicating those mAbs recognize Gβγ subunit as an epitope. Bottom right: Binding experiment with heterotrimeric Gi1

Despite exhaustive attempts to crystalize a complex between rhodopsin and heterotrimeric G_i1_^16^, we were unsuccessful in producing diffraction quality crystals. We presumed that this was due to the flexibility of the alpha-helical domain of Gα_i1_ as this domain separates from Ras-like domain and becomes flexible upon receptor-mediated activation in the nucleotide-free state^17,18^. We then set out to discover antibodies that could reduce this flexibility and facilitate crystallographic and cryo-EM structural studies of the complex. Mice were immunized with purified rhodopsin/G_i1_ complex and hybridoma cells were prepared from the isolated mice splenocytes. Clones that showed enzyme-linked immunosorbent assay (ELISA) and immunoprecipitation positive reaction were screened further using an analytical size-exclusion chromatography (SEC) assay with purified monoclonal antibodies. Most of the SEC-positive clones were Gβγ-binders, reflecting that it is the most stable and rigid component of the complex (Fig. 1a, c). Interestingly, we found a single clone that binds and confers GTPγS resistance to the rhodopsin/G_i1_ complex (Fig. 1a, b). Based on the clone identification number, we named its antibody mAb16. Notably, this clone does not show binding to any single component of G_i1_, but binds specifically to the intact heterotrimeric form of G_i1_, suggesting that it binds a composite epitope at the interface between Gα_i1_ and Gβγ subunits (Fig. 1c). We provide amino-acid sequence of mAb16 in the Supplementary Note 1.

### Crystal structure of Gi1/scFv16

**Fig. 2:**
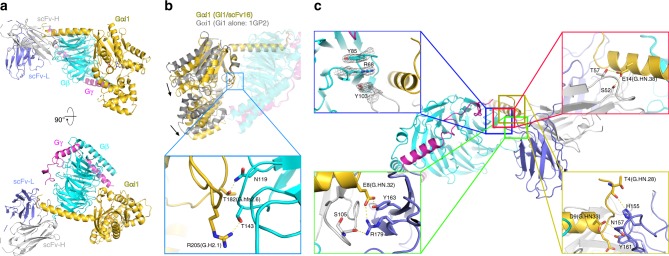
Crystal structure of G_i1_/scFv16 and characterization of Fab16. **a** Overall structure of Gi1/scFv16 complex. Cartoon representation with Gα_i1_ in gold, Gβ in cyan, Gγ in magenta, scFv-heavy chain in light grey and scFv-light chain in light blue. **b** Superposition of G_i1_/scFv16 structure onto G_i1_ (PDB: 1GP2) based on alignment of Gβγ subunits. Gα_i1_ (1GP2) in grey and G_i1_/scFv16 in the same colour code as in **a**. For clarity, Gβγ subunits and scFv16 is shown as transparent cartoon. Arrows show a slight rotational displacement of Gα_i1_ towards Gβ_1_ compared to Gi1 alone. Additional interactions are formed between switch I and switch II of Gα_i1_ and Gβ_1_. **c** Interaction between G_i1_ and scFv16. The residues participating in the interactions are depicted with stick models in the expanded panels. Residue numbers are shown with Common Gα Numbering (CGN) code for Gα_i1_^42^

In order to better understand the recognition mode of mAb16, we crystallized a fully soluble heterotrimeric G_i1_ in complex with mAb16 fragments. We tried both a Fab fragment (Fab16) and single-chain variable fragment (scFv16) derived from mAb16. Both Gi1/Fab16 and Gi1/scFv16 complexes formed crystals but only the scFv16 version diffracted to high resolution, presumably due to the intrinsic flexibility of the linker between the variable and the constant domain of the Fab^19^. The crystal structure of the G_i1_/scFv16 complex was solved at 2.0 Å by molecular replacement using G_i1_ (PDB ID: 1GP2) and an scFv fragment (PDB ID: 4NKD) as search models (Table 1). The overall structure of G_i1_ in complex with scFv16 is very similar to G_i1_ alone (Fig. 2a). The relative position of Ras-like domain and alpha-helical domain of Gα_i1_ moves closer to Gβ_1_ by a small rotation movement around the αN-b1 junction (Fig. 2b). This slight movement leads to two additional interactions between Thr182 of Gαi1 and Asn119 of Gβ_1_, and Arg205 of Gαi1 and Thr143 of Gβ_1_, located in Switch I and Switch II region, respectively (Fig. 2b). This could be due to the tighter association between these two subunits mediated by scFv16, although it may be the consequence of different crystal contacts between G_i1_ alone and G_i1_/scFv16. The structure of the G_i1_/scFv16 complex shows that scFv16 recognizes an epitope composed of the terminal part of the αN helix of Gα_i1_ as well as part of the Gβ_1_ subunit (Fig. 2c, Supplementary Fig. 1). The complementarity-determining region 3 of the heavy chain (CDR-H3) extends to interact with Gβ_1_ with its tip and Gα_i1_ with its side. CDR2-H2 and CDR-H1 support the interaction with Gα_i1_ and Gβ_1_, respectively by making hydrogen bonds and van der Waals contacts. CDR-L1 is exceptionally long and makes extensive contact with the edge of the αN helix together with CDR-L3 (Fig. 2c). There is no obvious interaction between scFv16 and Gγ_2_ subunit.

**Table 1:** Data collection and refinement statistics

	Gi1/scFv16^a^
*Data collection*	
Space group	*P*222_1_
Cell dimensions (σ)	
*a*, *b*, *c* (Å)	58.51, 104.74, 211.82
*α*, *β*, *γ* (°)	90.00, 90.00, 90.00
Resolution (Å)	39.26–2.00(2.07–2.00)^b^
*R*_sym_ or *R*_merge_	0.176(0.888)
*I/σI*	9.91(0.73)
Completeness (%)	99.16(99.47)
Redundancy	4.6(4.8)
*Refinement*	
Resolution (Å)	39.26–2.00(2.07–2.00)
No. reflections	88,191(8710)
*R*_work_/*R*_free_	0.1746/0.2097(0.2682/0.2940)
No. atoms	
Protein	7567
Ligand/ion	41
Water	628
*B*-factors (Å^2^)	
Protein	47.08
Ligand/ion	31.55
Water	51.44
R.m.s. deviations	
Bond lengths (Å)	0.007
Bond angles (°)	1.19

^a^The data set was collected from one single crystal

^b^Values in parentheses are for highest-resolution shell

**Fig. 3:**
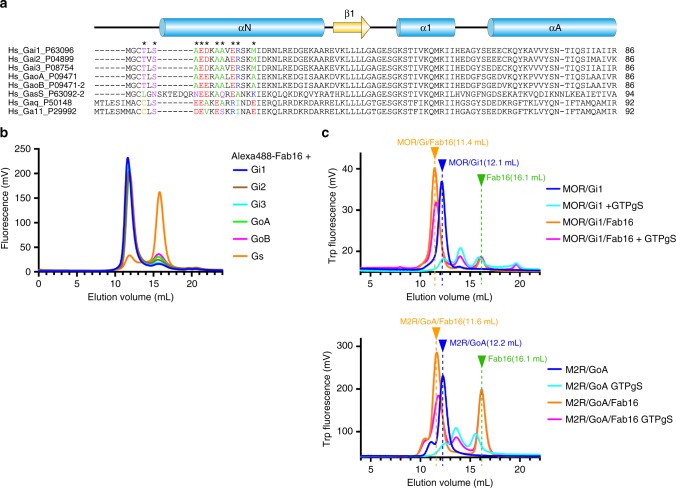
Sequence alignment of G-protein family members and binding profile of Fab16. **a** Multiple sequence alignment of amino-termini of representative Gα subunits from human. UniProt numbers are provided after each G-protein subtype name. Secondary structures are shown as cylinder (helix) and arrow (strand). The asterisks indicate the residues in contact with scFv16 in Gα_i1_ and those corresponding residues are coloured according to their property: Positive in blue, negative in red, hydrophobic in green, polar in purple, cysteine in yellow. **b** Fluorescent SEC analysis of binding of the fluorescently labelled Fab16 with G-protein family members. **c** Analytical tryptophane fluorescent SEC of μOR/G_i1_ and M_2_R/G_oA_ with GTPγS in the presence or absence of Fab16. Each complex alone runs around 12.2 mL. Upon binding to Fab16, they run at 11.4 mL or 11.6 mL indicating the binding of Fab16 to these GPCR/G-protein complexes. Excess free Fab16 runs at 16.1 mL. Dissociated components upon incubating with GTPγS show smaller peaks at 13.5–16 mL

As the α subunits of all G_i/o_ family members have high sequence similarity at the epitope residues in the αN helix (Fig. 3a) and can form a complex with Gβ_1_γ_2_, we expected that Fab16 would bind to all G_i/o_ family proteins. Using analytical fluorescent SEC, we show that Fab16 can bind to five different G_i/o_ type G-proteins but not G_s_, as it has poor sequence similarity to G_i/o_ members at this epitope region (Fig. 3b).

### Application to other GPCR-G_i/o_ complexes

Since Fab16 was initially isolated as a stabilizing agent that confers resistance to GTPγS triggered dissociation to a rhodopsin/G_i1_ complex and was later found to bind to a panel of G_i/o_ family members, we investigated whether it confers the same GTPγS resistance to other G_i/o_ type GPCR complexes. We chose a μ-opioid receptor/G_i1_ (μOR/G_i1_) and an M_2_ muscarinic acetylcholine receptor/G_oA_ (M_2_R/G_oA_) as representative family A G_i/o_-coupling GPCR complexes. Purified μOR/G_i1_ or M_2_R/G_oA_ complex solubilized in detergent was incubated with GTPγS in the presence or absence of Fab16, then analysed for dissociation by analytical SEC. Both μOR/G_i1_ and M_2_R/G_oA_ complexes showed a leftward peak shift upon incubating with Fab16, indicating its binding and also became GTPγS resistant, as they showed much less dissociation in the presence of Fab16 than the complex alone (Fig. 3c). These data with GPCR/G_i/o_ complexes are consistent with the binding experiments of Fab16 with G-protein alone, and indicate that it stabilizes G_i/o_ type GPCR complexes in general. In order to show the applicability of scFv16 to structural analysis of GPCR-G protein complexes, we have recently solved a near-atomic resolution map of μOR/G_i1_ complex using scFv16^15^. The presence of scFv16 enhanced complex stability towards specimen vitrification for cryo-EM, thereby enabling quality single-particle reconstructions.

### Influence of Fab16 on nucleotide binding

**Fig. 4:**
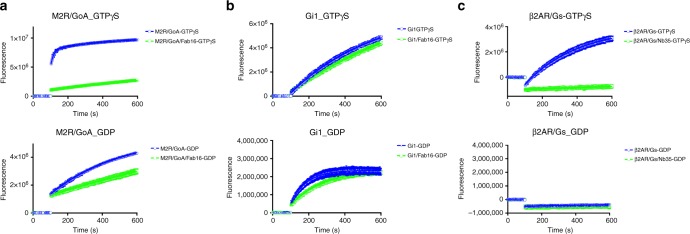
Nucleotide-binding kinetics. **a** Influence of Fab16 on the nucleotide-binding kinetics of the purified M_2_R/G_oA_ complex. Nucleotide binding was monitored by using BODIPY-FL-GTPγS or BODIPY-FL-GDP. **b** Influence of Fab16 on the nucleotide release kinetics of G_i1_. GDP release was monitored by BODIPY-FL-GTPγS- or BODIPY-FL-GDP-binding kinetics under conditions where GDP release is the rate-limiting step. **c** Nucleotide binding to β2AR/G_s_ complex in the presence or absence of Nb35. The curves represent the mean ± standard error of three experiments

In order to further investigate mAb16 for its protection mechanism against GTPγS, we monitored the binding kinetics of GTPγS to nucleotide-free M_2_R/G_oA_ complex. In the absence of Fab16, BODIPY-FL-GTPγS, a fluorescent analogue of GTPγS, binds to the complex with fast kinetics reflecting its ability to bind and trigger the dissociation of the complex. In contrast, BODIPY-FL-GTPγS binds to M_2_R/G_oA_/Fab16 complex ~70 times slower and to a much lower extent (Fig. 4a). On the other hand, the binding of GDP is only modestly affected when the complex is bound to Fab16 (Fig. 4a). Next we examined the basal nucleotide exchange rate of Gi1 and Gi1/Fab16 under conditions where GDP release is rate-limiting (Fig. 4b). Fab16-bound Gi1 releases GDP ~1.5 to 2-fold slower than Gi1 alone (Table 2). These findings suggest that in the absence of a coupled GPCR, Fab16 stabilizes the heterotrimer in its GDP-bound conformation. As a comparison, we examined the effect of Nb35 that was originally developed against the β_2_AR/G_s_ complex^2^. Structurally, it binds at the interface between switch II and helix III of Gαs and Gβ_1_, and has been characterized to provide GTPγS resistance to a GPCR/G_s_ complex. It’s been used for structural studies of all GPCR/G_s_-type complexes both in crystallography and cryo-EM so far^2,5–7^. We monitored the binding kinetics of GTPγS and GDP to the nucleotide-free β_2_AR/G_s_ complex. BODIPY-FL-GTPγS binds to the complex with fast kinetics in the absence of Nb35 but becomes extremely slow or negligible when bound with Nb35 (Fig. 4c), presumably due to the inhibition of the conformational change in switch II and switch III. BODIPY-FL-GDP showed no detectable binding to the complex alone or to the complex bound with Nb35 (Fig. 4c).

**Table 2:** Effect of Fab16 or Nb35 on the nucleotide-binding/releasing rate

	**M2R/GoA** **+** **GTPγS**	**M2R/GoA** **+** **Fab16** **+** **GTPγS**	**M2R/GoA** **+** **GDP**	**M2R/GoA** **+** **Fab16** **+** **GDP**
*k* (1/*s*)	0.061 ± 0.002 (fast)^a^	0.00092 ± 0.00004	0.00244 ± 0.00003	0.00042 ± 0.00009
*k* (1/*s*)	0.0032 ± 0.0002 (slow)^a^			
	**Gi1** **+** **GTPγS**	**Gi1** **+** **Fab16** **+** **GTPγS**	**Gi1** **+** **GDP**	**Gi1** **+** **Gab16** **+** **GDP**
*k* (1/*s*)	0.00206 ± 0.00003	0.00116 ± 0.00003	0.0115 ± 0.0002	0.00572 ± 0.00007
	**β**_**2**_**AR/G**_**s**_ **+** **GTPγS**	**β**_**2**_**AR/G**_**s**_ **+** **Nb35** **+** **GTPγS**	**β**_**2**_**AR/G**_**s**_ **+** **GDP**	**β**_**2**_**AR/G**_**s**_ **+** **Nb35** **+** **GDP**
*k* (1/*s*)	0.00327 ± 0.00005	ND	ND	ND

All values are expressed as mean ± s.e.m. of triplicate experiments

^a^The binding kinetics was best fit and analysed by two phase association. The fraction corresponding to the fast component is 0.526 ± 0.005

### Generalization of mAb16 binding to other G-protein subtypes

**Fig. 5:**
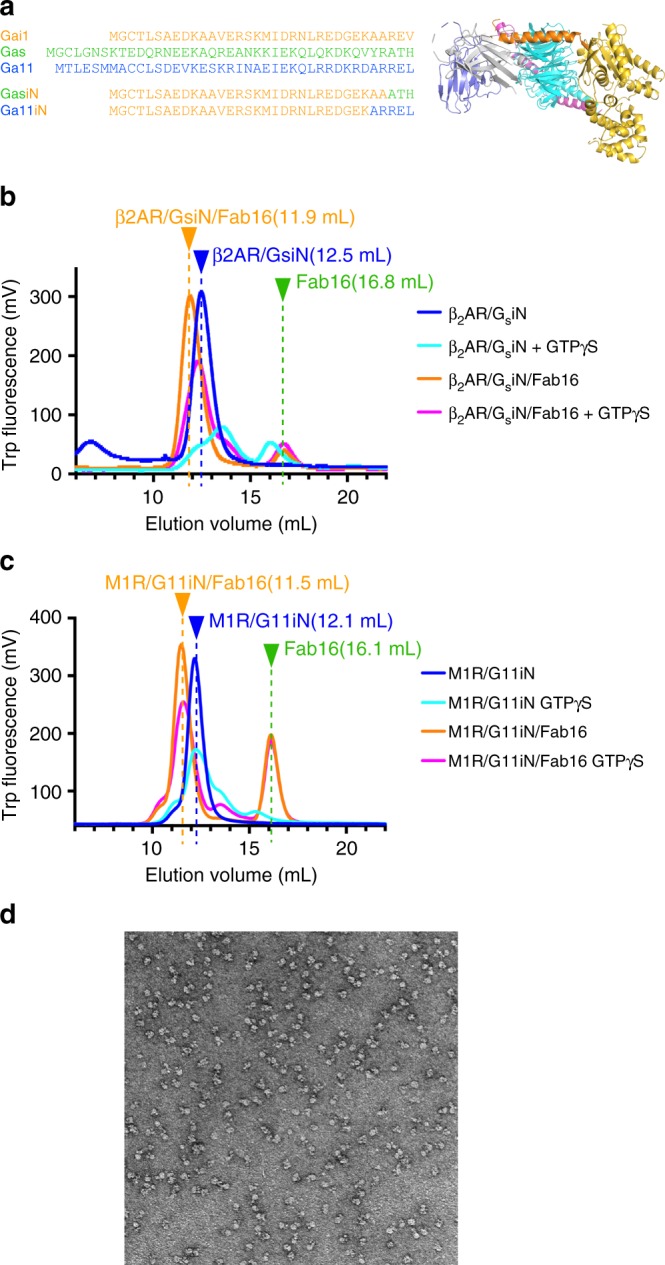
Generation of chimeric G-proteins. **a** Alignment of the αN helix of the G-protein subfamilies and the sequence of the chimeric Gα subunits. Transferred region from Gα_i1_ in each chimera is colored in orange. **b**, **c** Analytical SEC of β2AR/G_siN_ and M_1_R/G_11iN_ complexes incubated with GTPγS in the presence or absence of Fab16. Protein elution profiles were monitored by the intrinsic tryptophan fluorescence. **d** Negative stain electron microscopy image of purified the M_1_R/G_11iN_/scFv16 complex

Because mAb16’s epitope is located on a short stretch of the αN helix of the Gα subunit (Figs. 2a, b and 3a) as well as a small part of the Gβ_1_ subunit that can complex with all Gα-protein subtypes, we sought to engineer the αN helix of the other G-protein α-subunits in order to generalize scFv16 binding to all G-protein subtypes. Starting with the Gα_s_ subunit, we generated a chimera in which the αN helix was replaced by the equivalent region of Gα_i_ (Gα_si_N; residues 1–38 of Gαs replaced by residues 1–31 of Gαi1) (Fig. 5a). The Gα_si_N protein forms a heterotrimer with the Gβγ subunit and forms a stable complex with the β_2_AR (Fig. 5b). The β_2_AR/G_si_N complex can bind Fab16 and is largely protected from dissociation induced by GTPγS, whereas β2AR/GsiN complex alone dissociated almost completely under the same condition (Fig. 5b). We then used the same engineering approach to transfer Fab16 binding ability to G_11,_ a G_q_ family member, and replaced the αN helix of Gα_11_ with that from Gα_i1_ (Gα_11i_N, residues 1–35 of Gα_11_ replaced by residues 1–29 of Gα_i1_) (Fig. 5a). The Gα_11i_N protein forms a heterotrimer with Gβγ subunit and couples to form a stable complex with the M_1_ muscarinic acetylcholine receptor (M_1_R) (Fig. 5c). The M_1_R/G_11i_N complex alone dissociates upon incubation with GTPγS, whereas the M_1_R/G_11i_N complex bound to Fab16 showed GTPγS resistance (Fig. 5c) consistent with G_i/o_ and G_si_N complexes. Negative stain EM visualization of the M_1_R/G_11i_N complex reveals a mono-disperse sample (Fig. 5d). These results demonstrate that Fab16 (or scFv16) can be used as a tool with broad variety of GPCR/G-protein complexes by substituting the αN helix of other Gα-subunit with equivalent region of Gαi1. Scanning the chimera junction between Gα_11_ and Gα_i1_ shows that a smaller substitution in the middle of the αN helix is still tolerated for the expression and the heterotrimer formation (Supplementary Fig. 2a). When replaced with the equivalent residues with this minimal chimeric region (residues 1–18 of Gαi1), both G_s_ and G_11_ are enabled to bind Fab16 and form stable complexes with respective GPCRs (Supplementary Fig. 2b, c.) The same minimal region when transferred to G_12_ also enables Fab16 binding (Supplementary Fig. 2d). The binding interface of Gβ1 subunit to scFv16 is limited compared to Gα subunit in the crystal structure. These residues are mostly conserved among Gβ family members except Gβ5 (Supplementary Fig. 3). There is no direct interaction between Gγ2 and scFv16 in the crystal structure. The fact that mAb16 was originally raised against and indeed binds to Rhodopsin/Gi1 that is composed of Gγ1 from the native bovine retina and still binds G-proteins or GPCR/G-protein complexes composed of Gγ2 indicates that Fab/scFv16 binds to the heterotrimeric G-protein regardless of the composition of the γ-subunit. Therefore, the binding ability to Fab16 is transferable to broad range of G-protein family members with minimal chimeric constructs. We provide amino-acid sequences of G-protein chimera constructs in the Supplementary Note 2 as well as the primers used for the construction in the Supplementary Table.

## Discussion

In this work, we have developed a unique antibody fragment that recognizes an interface on heterotrimeric G_i1_. The antibody confers the GPCR–G_i/o_ complexes the resistance to GTPγS-induced dissociation. This property is also observed with formerly identified Nb35 for Gs complexes^2^. Nb35 and mAb16 engage distinct epitopes at the G-protein interface: Nb35 binds at the switch II and αIII helix of Gα_s_ and Gβ, while mAb16 engages the αN helix of Gα_i1_ and Gβ. The switch II region adopts a distinct conformation upon binding of GTPγS compared to the nucleotide-free or GDP-bound state observed in the crystal structures as well as in the EPR spectroscopic measurement^2,20–23^. Nb35 is reported to suppress nucleotide exchange turnover of the CTR/G_s_ complex^5^ and indeed it prevents GTPγS binding to the β2AR/G_s_ complex (Fig. 4c), which we presume due to the fixed conformation of the switch II in the nucleotide-free state and steric clash of the switch III with Nb35. On the other hand, mAb16 binds 40 Å away from the nucleotide-binding pocket with no direct contact with this region yet prevents the binding of GTPγS to the nucleotide-free GPCR/G-protein complex (Fig. 4a) and helps heterotrimeric G_i1_ to retain GDP in the nucleotide-binding pocket (Fig. 4b). It has been reported that heterotrimer formation rigidifies the switch II region that is in direct contact with Gβ subunit^23^. Thus, by stabilizing interactions between the Gβ subunit and switch II, mAb16 prevents GTPγS binding to the empty pocket. This may also explain the slower GDP release as Gβ subunit functions as a GDP dissociation inhibitor (GDI)^24^. Additional interactions formed between switch I, II and Gβ_1_ subunit upon scFv16 binding supports this idea (Fig. 2b). Another possibility would be inferred from HDX-MS measurements that revealed the dynamic nature of αN-β1 junction of G_s_ during the complex formation with β2AR and the dissociation upon addition of GDP/AlF4^25^. Binding of mAb16 would tighten the association of αN helix with Gβ subunit and concomitantly reduce the dynamics of αN–β1 junction, which may influence the binding of nucleotide through the β1-strand and the P-loop that is in direct contact with nucleotide. Previous work showing that binding of GTPγS triggers release of αN helix from Gβ and promotes its unfolding^26^ indicates that there could be an allosteric effect between the nucleotide-binding pocket and αN helix.

Contrary to the large diversity of different GPCR genes in the human genome, there are only four major G-protein family members: G_s_, G_i/o_, G_q/11_ and G_12/13_^27^. Among them G_i/o_ is the most broadly coupling G-protein^10^. mAb16 is originally raised against rhodopsin/G_i1_ complex and binds rhodopsin/G_i1_, μOR/G_i1_ and M_2_R/G_oA_ complexes, suggesting that it likely binds all G_i/o_ type complexes, according to the sequence similarity of the epitope residues and the binding profile of individual G-protein alone (Fig. 3b). In contrast, the binding interface of Gα_s_ to Nb35 is not conserved among Gα subtypes; therefore it would require elaborate protein engineering to transfer the binding surface to other family members or to evolve the nanobody itself. On the other hand, the binding surface of Gα_i1_ to mAb16 is located in a small stretch of the αN helix. Since the αN helix apparently serves as a separate module from the Ras-like domain to interact with Gβγ, it is more amenable to generating functional chimeras^28–30^. Previous studies have shown that the entire αN helix of Gα_q_, Gα_12_ and Gα_13_ can be substituted with the corresponding region of Gα_i1_ to produce functional chimeric G_i/q_, G_i/12_ and G_i/13_. These engineered G proteins retained the biochemical properties of their counterpart wild types, while αN helix of Gα_12/13_ is reportedly important for the receptor selectivity^31^. Chimeric Gs protein has also been made with various lengths of αN from Gαi subunit to investigate the functional role of this region^32,33^. While the chimeras exhibit a large constitutive activity when the substitution goes beyond αN helix to replace the residues 1–62 of Gα_s_ by 1–54 of Gα_i_, a minor increase in the basal activity was observed when residues 1–41 of Gα_s_ is replaced by 1–34 of Gα_i_. Our substitutions of G_siN_ and G_11iN_ are both within the range of these chimeric designs and therefore would be expected to behave in the same way as the wild-type counterparts. In fact, our G protein chimeras form functional heterotrimers with co-expressed Gβγ subunit and form stable and functional complexes with cognate GPCRs. The more conserved chimeras where the residues 1–18 of Gα_i1_ is transferred to the equivalent residues of Gα_s_, Gα_11_ and Gα_12_ are also able to bind with Fab16 (Supplementary Fig. 2).

In conclusion, the antibody fragment derived from mAb16 promotes the stabilization of GPCR/G-protein complexes and adds an asymmetric feature that may aid with cryo-EM particle projection alignment. The usefulness of the antibody fragment in structural determination was proven by the cryo-EM structure of μOR/G_i1_ complex where the presence of scFv16 stabilized the complex for high-resolution cryo-EM work. Furthermore, this antibody fragment can be applied to other G-protein subtypes with minimal protein engineering and therefore would be expected to be a broadly applicable tool for cryo-EM studies of any GPCR/G-protein complex.

## Methods

### Protein expression and purification

Rhodopsin/G_i1_ complex was purified as described previously^16^. Briefly, bovine rhodopsin with three mutations, N2C, M257Y and N282C, was stably expressed in and purified from HEK293S GnTI^−^ cells using 1D4 immunoaffinity chromatography. Purified rhodopsin was incubated with G_i1_ reconstituted from recombinant Gαi1 subunit from *Escherichia coli* BL21 (DE3) cells (Novagen) and Gβγ subunit purified from bovine retina (W L Lawson Company). Rhodopsin/G_i1_ complex formation was triggered by the irradiation through 495 nm long-pass filter in the presence of apyrase (Sigma-Aldrich). Rhodopsin/G_i1_ complex was separated from the free rhodopsin or G_i1_ by SEC on a Tricorn 10/600 column packed with Superdex 200 (GE healthcare) in a buffer containing 100 mM NaCl, 20 mM Hepes pH 7.5, 0.01% lauryl maltose neopentyl glycol (MNG), 2 mM 2-mercaptoethanol.

µOR with a cleavable amino and carboxy-terminal FLAG- and His-tag^13^ was expressed in *Spodoptera frugiperda* Sf9 insect cells using baculovirus infection system (Expression Systems). Cells were solubilized in 1% n-dodecyl-β-d-maltoside (DDM) (Anatrace), 0.2% 5-cholesterol hemisuccinate (CHS) (Steraloids) and the soluble fraction was purified by Ni-chelating sepharose chromatography. The eluted protein was supplemented with 2 mM CaCl2, loaded onto M1 anti-FLAG immunoaffinity column (prepared in house) and washed with progressively lower concentrations of the antagonist naloxone (Sigma-Aldrich). Receptor was eluted in a buffer consisting of 100 mM NaCl, 20 mM Hepes pH 7.5, 0.1% DDM, 0.01% CHS with 50 nM naloxone, and further purified by SEC on a Superdex 200 10/300 column in a buffer containing 1 µM lofentanil (Tronto Research Chemicals) to exchange the ligand. Monomeric fractions were pooled, further supplemented with a twofold molar excess of lofentanil and concentrated to ~100 µM for complex formation.

β2AR was purified in the same way as described previously^34^. Briefly, Sf9 insect cells were lysed by osmotic shock prior to solubilization of the membrane fraction by DDM. Solubilized receptor was first purified by M1 anti-FLAG immunoaffinity chromatography, followed by alprenolol-sepharose chromatograpy (alprenolol-sepharose resin prepared in house) to isolate only functional receptor. Alprenolol-sepharose eluate was concentrated on M1 FLAG affinity resin, and then washed with ligand-free buffer for 1 h at room temperature to eliminate bound alprenolol. Receptor was eluted in a buffer consisting of 20 mM Hepes pH 7.5, 350 mM NaCl, 0.1 % DDM and 0.01% CHS and further purified by size-exclusion chromatography on a Superdex 200 10/300 column (GE Healthcare) in buffer containing 20 mM Hepes pH 7.5, 100 mM NaCl, 0.05 % DDM, 0.005% CHS and 1 µM BI-167107 (Boehringer-Ingelheim). The eluated receptor was concentrated to ~100 µM for complexing.

For M_1_R and M_2_R, we used constructs used in previous studies^12,35^ with some modifications. Briefly, T4L in the ICL3 of M_1_R was removed, and residues 219–232 and 345–354 were added to TM5 and TM6, respectively. The ICL3 of M_2_R was extended by 15 amino-acid residues from TM6. Primer sequences for these modifications are provided in the Supplementary Table. The amino-acid sequences for these receptors are provided in the Supplementary Note 2. These receptors were purified essentially in the same way as µOR with detergent exchange to MNG during M1 FLAG chromatography and using atropine (Sigma-Aldrich) and iperoxo (Sigma-Aldrich) in place of naloxone and lofentanil, respectively.

Heterotrimeric G-proteins were expressed and purified as previously described. Briefly, *Trichuplusia ni* (Hi5) insect cells (Expression Systems) were co-infected with two viruses, one encoding the wild-type human Gα subunit and another encoding the wild-type human β1γ2 subunits with an decahistidine tag inserted at the amino terminus of the β1 subunit with HRV-3C protease cleavable site. In the case of G_11iN_, additional virus encoding Ric8A was also co-infected. Cells were harvested 48 h post infection, lysed in hypotonic buffer and lipid-modified heterotrimeric G-protein was extracted in a buffer containing 1% sodium cholate (Sigma-Aldrich) and 0.05% DDM. The soluble fraction was purified using Ni-chelating sepharose chromatography, and the detergent was exchanged from cholate/DDM mixture to DDM alone. After elution, HRV-3C protease (in-house prepared) was added and the protein was dialyzed against a buffer containing 20 mM Hepes pH 7.5, 100 mM NaCl, 1 mM MgCl2, 0.05% DDM, 100 μM Tris(2-carboxyethyl)phosphine hydrochloride (TCEP) (Sigma-Aldrich), 10 μM GDP (Sigma-Aldrich). Cleaved heterotrimeric G-protein was further purified by reloading over Ni-sepharose resin. The flow through was collected and purified over a size-exclusion chromatography using a Superdex 200 10/300 column.

Soluble Gβγ subunit for crystallography was expressed and purified from Trichuplusia ni (Hi5) insect cells. Hi5 cells were infected with baculovirus encoding the human β1γ2 subunits with cysteine 68 of γ2 subunit mutated to serine (Gβ1γ2C68S) to remove the geranylgeranylation modification. A decahistidine tag was attached at the amino terminus of the β1 subunit with HRV-3C protease cleavable site. Cultures were harvested 48 h post infection. Cells were lysed in the lysis buffer (10 mM Tris pH 7.4, 5 mM 2-mercaptoethanol, 160 μg/mL benzamidine and 2.5 μg/mL leupeptin). Following to the centrifugation, the supernatant was incubated with Ni-chelating sepharose. The resin was first washed with a high salt buffer (20 mM Hepes pH 7.5, 500 mM NaCl, 20 mM imidazole and 2 mM 2-mercaptoethanol) then a low salt buffer (20 mM Hepes pH 7.5, 100 mM NaCl, 20 mM imidazole and 100 μM TCEP). The protein was eluted with an elution buffer (20 mM Hepes pH 7.5, 100 mM NaCl, 250 mM imidazole and 100 μM TCEP) and dialysed against 20 mM Hepes pH 7.5, 100 mM NaCl and 100 μM TCEP after adding HRC-3C protease to cleave amino-terminal His-tag. Gβ1γ2C68S was further purified by reloading over Ni-sepharose resin. The flow through was collected and purified by a size-exclusion chromatography using a Superdex 200 10/300 column.

The human Gα_i1_ protein for crystallography was expressed in *Escherichia coli* Rosetta2 (DE3) cells (Novagen) with an N-terminal octahistidine-tag and an HRV-3C protease recognition site. The culture was grown at 37 °C in TB medium. When the OD 600 reached 0.6, the protein expression was induced with 0.5 mM IPTG and further grown for 20 h at 24 °C. The cells were harvested by centrifugation, resuspended in the lysis buffer (50 mM Tris-HCl, pH 7.5, 100 mM NaCl, 10 mM imidazole, 0.1 mM PMSF, 10 μM GDP and 5 mM 2-mercaptoethanol). The resuspended cells were disrupted by sonication. Cell lysate was clarified by centrifugation and the supernatant was incubated with Ni-chelating sepharose equilibrated with the lysis buffer. The resin was first washed with the high salt buffer (20 mM Hepes pH 7.5, 500 mM NaCl, 1 mM MgCl_2_, 20 mM imidazole, 10 μM GDP and 2 mM 2-mercaptoethanol) then the low salt buffer (20 mM Hepes pH 7.5, 100 mM NaCl, 1 mM MgCl_2_, 20 mM imidazole, 10 μM GDP and 100 μM TCEP). The protein was eluted with the elution buffer (20 mM Hepes pH 7.5, 100 mM NaCl, 1 mM MgCl_2_, 250 mM imidazole, 10 μM GDP and 100 μM TCEP) and dialysed against 20 mM Hepes pH 7.5, 100 mM NaCl, 1 mM MgCl_2_, 10 μM GDP and 100 μM TCEP after adding HRC-3C protease to cleave amino-terminal tag. Cleaved Gα_i1_ was further purified by reloading over Ni-sepharose resin. The flow through was collected and purified over a size-exclusion chromatography using a Superdex 200 10/300 column.

GPCR/G-protein complex was prepared essentially in the same way as described previously using agonists lofentanil, iperoxo, BI-167107 for μOR, M_1_R and M_2_R, β2AR, respectively^2^. Briefly, receptor was mixed with 1.2–1.5 molar excess G-protein. Following the incubation at room temperature for 1 h, apyrase was added and the reaction mixture was transferred to 4 °C and further incubated for 4 h to overnight. Prior to loading M1 FLAG column, 1% MNG and 0.1% CHS was added. The MNG concentration was progressively lowered during M1 FLAG wash. FLAG eluted protein was further purified by size-exclusion chromatography on a Superdex 200 10/300 column.

### Monoclonal antibody production and characterization

For the antigen, rhodopsin–G_i1_ complex was stabilized by crosslink using BS-3 (ThermoFisher). Naval Medical Research Institute (NMRI) mice were immunized intraperitoneally with the emulsified antigen. (This study was carried out in strict accordance with the Rules and Regulations for the Protection of Animal Rights (Tierschutzverordnung) of the Swiss Bundesamt für Veterinärwesen. The protocol was ethically approved by the Ethikkommission beider Basel (Permit Number: 237/23523).) Mice with strong ELISA reaction to the antigen were killed and the spleen was removed. Isolated splenocytes were fused with the myeloma cell partner (PAI mouse myeloma cells, derived from P3-x63-AG8) using polyethylene glycol 1500 (Roche Diagnostics). The fusion mixture was plated into multi-well plates (Thermo Scientific Nunc MicroWell Cell Culture High Flange 96-Well Microplates) and clonal hybridomas were selected by growing in HAT medium supplemented with culture supernatant of mouse macrophages P388. IgG positive clones were screened by ELISA for reactivity against Rhodopsin/G_i1_ complex. Clones that showed a positive reaction in an ELISA assay and by immunoprecipitation were further characterized as monoclonal antibodies or Fab fragments. Initial SEC analysis using rhodopsin/G_i1_ or each component was carried out in 20 mM Hepes pH 7.5, 100 mM NaCl, 2 mM 2-mercaptethanol and 0.01% MNG using Superdex 200 10/200 column.

Coding regions of the heavy-chain (VH-CH1) and light-chain (VL-CL) of mAb16 were cloned into the modified pVL1392 vector where VH-CH1 and VL-CL both attached with GP67 secretion signal sequence were under polyhedron and p10 promoter regulation, respectively. Octahistidine-tag with HRV-3C protease cleavable site was attached to the carboxy-terminus of VH-CH1 for the purification. The single-chain variable fragment of mAb16 (scFv16) was cloned into a modified pVL1392 vector containing a GP67 secretion signal immediately prior to the amino terminus of the scFv16. Octahistidine-tag with HRV-3C protease cleavable site was attached to the carboxy-terminus.

Both Fab16 and scFv16 were expressed in secreted form from Trichuplusia ni Hi5 insect cells using the baculovirus infection method (Expression Systems), and purified by Ni-sepharose chromatography. Supernatant from baculovirus infected cells was pH balanced by addition of Tris pH 7.5. Chelating agents were quenched by addition of 1 mM nickel chloride and 5 mM calcium chloride and incubation with stirring for 1 h at 25 °C. Resulting precipitates were removed by centrifugation and the supernatant was loaded over Ni-sepharose chromatography column. The column was washed with a high salt buffer (20 mM Hepes pH 7.5, 500 mM NaCl and 20 mM imidazole) followed by a low salt buffer (20 mM Hepes pH 7.5, 100 mM NaCl and 20 mM imidazole). The protein was eluted with the elution buffer (20 mM Hepes pH 7.5, 100 mM NaCl and 250 mM imidazole) and the carboxy-terminal octahistidine tag was cleaved by incubation with HRV-3C protease during dialysis against a buffer consisting of 20 mM Hepes pH 7.5 and 100 mM NaCl. Cleaved protein was further purified by reloading over Ni-NTA resin. The flow through was collected and purified over a size-exclusion chromatography using a Superdex 200 16/60 column (GE healthcare). Monomeric fractions were pooled, concentrated and flash frozen in liquid nitrogen until use.

For the binding assay of Fab16 with heterotrimeric G-protein subtypes, Fab16 was first labelled with Alexa Fluor 488 NHS Ester (ThermoFisher Scientific) in 20 mM MES pH 6.5. Free dye was removed by G-50 desalting column (GE healthcare), and the labelled Fab16 was recovered and concentrated. 5–30 μM of G-protein was mixed with 0.4 μM of labelled Fab16, incubated for 1 h and run on SEC on a Superdex 200 10/300 column. Fluorescence signal was recorded with excitation at 488 nm and emission at 512 nm.

### Construction of chimeric G proteins

Gα_siN_ was constructed by substituting the residues 1–38 of Gα_s_ with the residues 1–31 of Gα_i1_. Gα_11iN_ were constructed by substituting the residues 1–36 of Gα_11_ with the residues 1–29 of Gα_i1_. These constructs were cloned into pFastBac1 vector and baculovirus were made according to the manufacturer. Scanning chimeras of Gα11 as well as Gα_siN18_, Gα_11iN18_ and Gα12_iN18_ are also made in the same way. Primer sequences used to construct the chimeras are provided in the Supplementary Table. The amino-acid sequences of the chimera constructs are provided in the Supplementary Note 2.

### Characterizing resistance to GTPγS

GTPγS resistance test was performed in the buffer containing 20 mM Hepes pH7.5, 100 mM NaCl, 100 μM TCEP, 0.01% MNG, with each agonist for the complex, lofentanil, iperoxo, BI167107 for μOR/G_i1_, M_1_R/G_11iN_ and M_2_R/G_oA_, β2AR/G_siN_, respectively. Purified complex with or without Fab16 was incubated with 100 μM GTPγS in the buffer and incubated for 1 h at 24 °C followed by SEC analysis on Superdex 200 10/300 monitoring the protein intrinsic fluorescence with the excitation wavelength at 280 nm and emission wavelength 340 nm.

### Determination of the structure of the G_i1_/scFv16 complex

For G_i1_/scFv16 crystallization, separately purified and concentrated Gα_i1_, Gβ1γ2C68S and scFv16 were mixed at a 1:1:1.2 molar ratio and incubated for 30 min at 24 °C. The resulting G_i1_/scFv16 complex was purified from uncomplexed subunits and free scFv16 by SEC in the buffer containing 20 mM Hepes pH7.5, 100 mM NaCl, 1 mM MgCl2, 10 μM GDP and 100 μM TCEP. Purified G_i1_/scFv16 was incubated with 1 mM aluminium chloride and 50 mM sodium fluoride for 1 h on ice, concentrated to 10–15 mg/mL and crystallized using the hanging drop vapour diffusion method at 20 °C against a reservoir solution containing 10% PEG 8000, 0.1 M Sodium citrate pH 5.0, 1 mM MgCl_2_, 10 μM GDP, 100 μM TCEP, 1 mM aluminium chloride and 10 mM sodium fluoride. Crystals appeared within a few hours and grew to the full size in 5 days. Crystals were soaked into the reservoir solution supplemented with 25% glycerol as a cryo-protectant and flash frozen in liquid nitrogen. The X-ray data set was collected at the experimental station 12-2 in the Stanford Synchrotron Radiation Lightsource. Diffraction data were integrated by XDS^36^, scaled and merged by AIMLESS^37^. The structure was solved by the molecular replacement in Phaser^38^ using the heterotrimeric Gi-protein (1GP2) and scFv fragment (4NKD) as independent search models. Manual model building was performed in Coot^39^ and refinement was performed with Phenix refine^40,41^. Ramachandran statistics are favoured 96.6%, allowed 3.4%, outlier 0.0%.

### Nucleotide-binding studies

For the nucleotide-binding experiment, fluorescence from BODIPY-FL-GTPγS or BODIPY-FL-GDP (ThermoFisher Scientific) was recorded using the Fluorolog spectrophotometer (HORIBA) in the 500 μL quartz cuvette. The fluorophore was exited at 495 nm and emission was detected at 508 nm at 22 °C. The buffer composition is 20 mM HEPES, pH 7.5, 100 mM sodium chloride, 0.01% LMNG, 10 mM magnesium chloride, 100 μM TCEP and 10 μM iperoxo or BI-167107 for GPCR/G-protein complexes, and 20 mM HEPES, pH 7.5, 100 mM sodium chloride, 0.02% DDM, 10 mM magnesium chloride, 100 μM TCEP for Gi1. Kinetics data were collected with 1 μM fluorophore alone for 100 s to establish the baseline fluorescence intensity. Protein was added to 200 nM and rapidly mixed in the fluorescence cuvette. Data points were acquired every second for 600 s. The resulting kinetics spectra were plotted and fit to one phase- or two phase-association function using GraphPad Prism 7.0.

### Data availability

Data supporting the findings of this manuscript are available from the corresponding authors upon reasonable request. Structure and data set in this work have been deposited in the Protein Data Bank under accession code PDB 6CRK.

## Electronic supplementary material

Supplementary Information

Peer Review File
