# Supplementary material for: Development of an antibody fragment that stabilizes GPCR/G-protein complexes
Source: Nat Commun. 2018 Sep 13;9:3712. doi: 10.1038/s41467-018-06002-w (PMC6137068; doi:10.1038/s41467-018-06002-w)
Supplement: Supplementary file 1 — Supplementary Information [file 41467_2018_6002_MOESM1_ESM.pdf]

Supplementary Information

Development of an antibody fragment that stabilizes GPCR/G-protein complexes

Maeda et al.

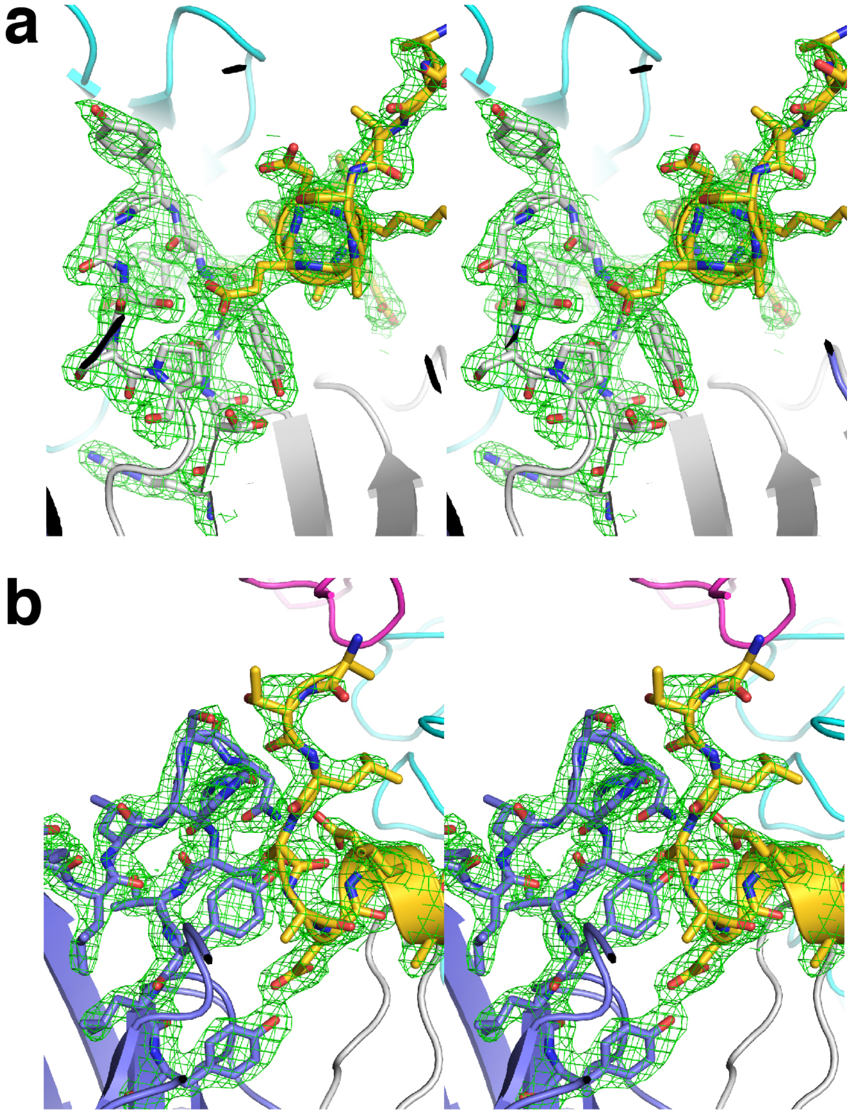

Supplementary Figure 1. Electron density map. Stereo view of the electron density map of **(a)** CDR-H3/αN helix interface and **(b)** CDR-L1/αN helix. The electron density is shown as green mesh contoured at 1.5σ. Color scheme is the same as in the main text, and the residues are described as cartoon and sticks in the density map.

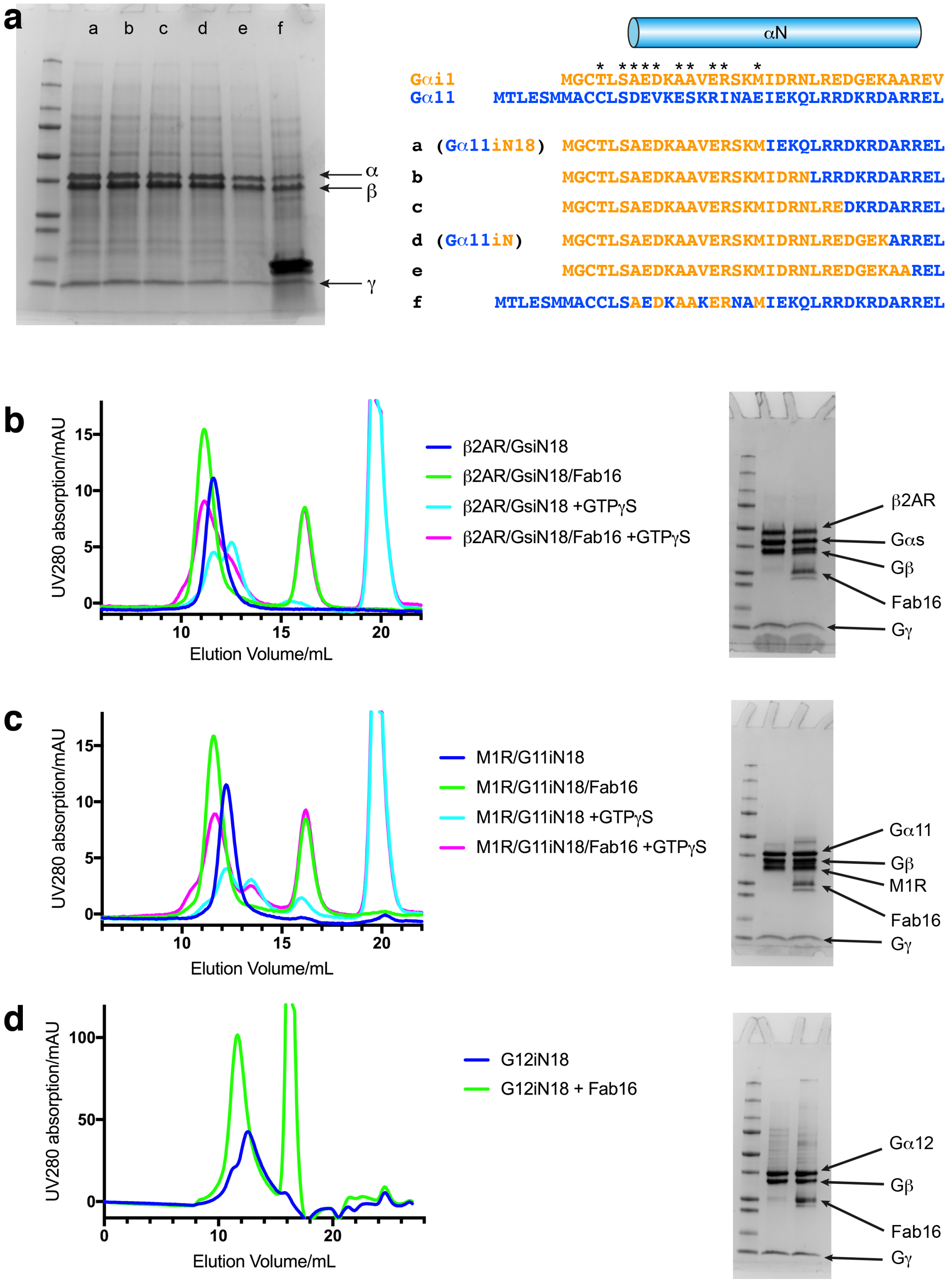

Supplementary Figure 2. Minimal chimera constructs for G_s, G_{11}, and G_{12}. (a) Left: Coomassie stained SDS-PAGE gel of chimera constructs co-expressed with Gβγ. Proteins were purified from the solubilized lysate by Ni-affinity chromatography. The bands corresponding each subunit are labeled. Right: Sequences of the designed

chimeras corresponding to the labels in the SDS-PAGE. Region of αN helix is shown as a cylinder and the residues interacting with scFv16 are marked with the asterisks. **(b, c)** Analytical SEC of β2AR/G_{siN18} and M_1R/G_{11iN18} complexes incubated with GTPγS in the presence or absence of Fab16. Coomassie-stained SDS-PAGE gels of peak fractions corresponding to the β2AR/G_{siN18} or M_1R/G_{11iN18} alone (middle lane), and β2AR/G_{siN18}/Fab16 or M_1R/G_{11iN18}/Fab16 (right lane). **(d)** Analytical SEC of G_{12iN18} in the presence or absence of Fab16. Coomassie-stained SDS-PAGE gels of peak fractions corresponding to G_{12iN18} alone (middle lane), and G_{12iN18} /Fab16 (right lane).

```
Gβ1   --------------------------------------------------MSELDQLRQE    10
Gβ2   --------------------------------------------------MSELEQLRQE    10
Gβ3   --------------------------------------------------MGEMEQLRQE    10
Gβ4   --------------------------------------------------MSELEQLRQE    10
Gβ5   MCDQTFLVNVFGSCDKCFKQRALRPVFKKSQQLSYCSTCAEIMATEGLHENETLASLKSE    60

Gβ1   AEQLKNQIRDARKACADATLSQITNNIDPVGRIQMRTRRTLRGHLAKIYAMHWGTDSRLL    70
Gβ2   AEQLRNQIRDARKACGDSTLTQITAGLDPVGRIQMRTRRTLRGHLAKIYAMHWGTDSRLL    70
Gβ3   AEQLKKQIADARKACADVTLAELVSGLEVVGRVQMRTRRTLRGHLAKIYAMHWATDSKLL    70
Gβ4   AEQLRNQIQDARKACNDATLVQITSNMDSVGRIQMRTRRTLRGHLAKIYAMHWGYDSRLL    70
Gβ5   AESLKGKLEEERAKLHDVELHQVAERVEALGQFVMKTRRTLKGHGNKVLCMDWCKDKRRI    120

Gβ1   VSASQDGKLIIWDSYTTNKVHAIPLRSSWVMTCAYAPSGNYVACGGLDNICSIYNLKTRE    130
Gβ2   VSASQDGKLIIWDSYTTNKVHAIPLRSSWVMTCAYAPSGNFVACGGLDNICSIYSLKTRE    130
Gβ3   VSASQDGKLIVWDSYTTNKVHAIPLRSSWVMTCAYAPSGNFVACGGLDNMCSIYNLKSRE    130
Gβ4   VSASQDGKLIIWDSYTTNKMHAIPLRSSWVMTCAYAPSGNYVACGGLDNICSIYNLKTRE    130
Gβ5   VSSSQDGKVIVWDSFTTNKEHAVTMPCTWVMACAYAPSGCAIACGGLDNKCSVYPLTFDK    180

Gβ1   GN--VRVSRELAGHTGYLSCCRFLD-DNQIVTSSGDTTCALWDIETGQQTTTFTGHTGDV    187
Gβ2   GN--VRVSRELPGHTGYLSCCRFLD-DNQIITSSGDTTCALWDIETGQQTVGFAGHSGDV    187
Gβ3   GN--VKVSRELSAHTGYLSCCRFLD-DNNIVTSSGDTTCALWDIETGQQKTVFVGHTGDC    187
Gβ4   GN--VRVSRELPGHTGYLSCCRFLD-DSQIVTSSGDTTCALWDIETAQQTTTFTGHSGDV    187
Gβ5   NENMAAKKKSVAMHTNYLSACSFTNSDMQILTASGDGTCALWDVESGQLLQSFHGHGADV    240

Gβ1   MSLSLAPD--TRLFVSGACDASAKLWDVREGMCRQTFTGHESDINAICFFPNGNAFATGS    245
Gβ2   MSLSLAPD--GRTFVSGACDASIKLWDVRDSMCRQTFIGHESDINAVAFFPNGYAFTTGS    245
Gβ3   MSLAVSPD--FNLFISGACDASAKLWDVREGTCRQTFTGHESDINAICFFPNGEAICTGS    245
Gβ4   MSLSLSPD--MRTFVSGACDASSKLWDIRDGMCRQSFTGHVSDINAVSFFPNGYAFATGS    245
Gβ5   LCLDLAPSETGNTFVSGGCDKKAMVWDMRSGQCVQAFETHESDINSVRYYPSGDAFASGS    300

Gβ1   DDATCRLFDLRADQELMTYSHDNIICGITSVSFSKSGRLLLAGYDDFNCNVWDALKADRA    305
Gβ2   DDATCRLFDLRADQELLMYSHDNIICGITSVAFSRSGRLLLAGYDDFNCNIWDAMKGDRA    305
Gβ3   DDASCRLFDLRADQELICFSHESIICGITSVAFSLSGRLLFAGYDDFNCNVWDSMKSERV    305
Gβ4   DDATCRLFDLRADQELLLYSHDNIICGITSVAFSKSGRLLLAGYDDFNCNVWDTLKGDRA    305
Gβ5   DDATCRLYDLRADREVAIYSKESIIFGASSVDFSLSGRLLFAGYNDYTINVWDVLKGSRV    360

Gβ1   GVLAGHDNRVSCLGVTDDGMAVATGSWDSFLKIWN    340
Gβ2   GVLAGHDNRVSCLGVTDDGMAVATGSWDSFLKIWN    340
Gβ3   GILSGHDNRVSCLGVTADGMAVATGSWDSFLKIWN    340
Gβ4   GVLAGHDNRVSCLGVTDDGMAVATGSWDSFLRIWN    340
Gβ5   SILFGHENRVSTLRVSPDGTAFCSGSWDHTLRVWA    395
```

Supplementary Figure 3. Sequence alignment of G-protein β-subunits. Multiple sequence alignment of Gβ subunit from human. The residues interacting with scFv16 in the crystal structure were shown with the black background.

Heavy chain

```
AB16_HC  MDSRLNLVFL VLTLKGVQCD VQLVESGGGL VQPGGSRKLS CSASGFAFSS FGMHWVRQAP   60
         --leader sequence---                                 CDR1
AB16_HC  EKGLEWVAYI SSGSGTIYYA DTVKGRFTIS RDDPKNTLFL QMTSLRSEDT AMYYCVRSIY  120
                     CDR2
AB16_HC  YYGSSPFDF WGQGTTLTVS SAKTTPPSVY PLAPGCGDTT GSSVTLGCLV KGYFPESVTV  179
           CDR3               ---constant region-----------------------
AB16_HC  SGPISTINPC PPCKECHKCP APNLEGGPSV FIFPPNIKDV LMISLTPKVT CVVVDVSEDD  299
         --------------------------constant region------------------------
AB16_HC  PDVQISWFVN NVEVHTAQTQ THREDYNSTI RVVSTLPIQH QDWMSGKEFK CKVNNKDLPS  359
         --------------------------constant region------------------------
AB16_HC  PIERTISKIK GLVRAPQVYI LPPPAEQLSR KDVSLTCLVV GFNPGDISVE WTSNGHTEEN  419
         --------------------------constant region------------------------
AB16_HC  YKDTAPVLDS DGSYFIYSKL NMKTSKWEKT DSFSCNVRHE GLKNYYLKKT ISRSPGK  476
         --------------------------constant region---------------------
```

Light chain

```
AB16_LC  MRCLAEFLGL LVLWIPGAIG DIVMTQATSS VPVTPGESVS ISCRSSKSLL HSNGNTYLY   59
         ---leader sequence---                         -------CDR1------
AB16_LC  WFLQRPGQSP QLLIYRMSNL ASGVPDRFSG SGSGTAFTLT ISRLEAEDVG VYYCMQHLEY  119
                          --CDR2--                              --CDR3
AB16_LC  PLTFGAGTKL ELKRADAAPT VSIFPPSSEQ LTSGGASVVC FLNNFYPKDI NVKWKIDGSE  179
         CDR3          -------------constant region-----------------------
AB16_LC  RQNGVLNSWT DQDSKDSTYS MSSTLTLTKD EYERHNSYTC EATHKTSTSP IVKSFNRNEC  239
         ---------------------------constant region-----------------------
```

Supplementary Note 1. Amino-acid sequence of mAb16. Sequence of Heavy- and light-chain of mAb16 is provided. Leader sequence, CDRs, constant region is indicated under the amino-acid sequence.

Supplementary Note 2 Amino acid sequences of the constructs used in the study

HA signal sequence highlighted in purple
FLAG-tag highlighted in cyan
His-tag highlighted in green
ICL3 highlighted in grey
Sequences from Gαi1 colored in orange

M1R
MKTIIALSYIFCLVFADYKDDDDAAAQTSAPPAVSPQITVLAPGKGPWQVAFIGI
TTGLLSLATVTGNLLVLISFKVNTELKTVNNYFLLSLACADLIIGTFSMNLYTTY
LLMGHWALGTLACDLWLALDYVASQASVMNLLLISFDRYFSVTRPLSYRAKR
TPRRAALMIGLAWLVSFVLWAPAILFWQYLVGERTVLAGQCYIQFLSQPIITFG
TAMAAFYLPVTVMCTLYWRIYRETENRARELAALQGSETPGGKEQLAKRKTFS
LVKEKKAARTLSAILLAFILTWTPYNIMVLVSTFCKDCVPETLWELGYWLCYV
NSTINPMCYALCNKAFRDTFRLLLLCRWDKRRWRKIPKRPGSVHRTPSRQCHH
HHHH*

M2R
MKTIIALSYIFCLVFADYKDDDDASTDSSDNSLALTSPYLEKTFEVVFIVLVAGS
LSLVTIIGNILVMVSIKVNRHLQTVNNYFLFSLACADLIIGVFSMNLYTLYTVIGY
WPLGPVVCDLWLALDYVVSNASVMNLLIISFDRYFCVTKPLTYPVKRTTKMAG
MMIAAAWVLSFILWAPAILFWQFIVGVRTVEDGECYIQFFSNAAVTFGTAIAAF
YLPVIIMTVLYWHISRASKSRIKKDKKEPVANQDPVSIVARKIVKMTKQPAKKK
PPPSREKKVTRTILAILLAFIITWAPYNVMVLINTFCAPCIPNTVWTIGYWLCYIN
STINPACYALCNATFKKTFKHLLMCHYKNIGATRPAGHHHHHHHH*

GαsiN
MGCTLSAEDKAAVERSKMIDRNLREDGEKAAATHRLLLLGAGESGKSTIVKQ
MRILHVNGFNGDSEKATKVQDIKNNLKEAIETIVAAMSNLVPPVELANPENQFR
VDYILSVMNVPDFDFPPEFYEHAKALWEDEGVRACYERSNEYQLIDCAQYFLD
KIDVIKQADYVPSDQDLLRCRVLTSGIFETKFQVDKVNFHMFDVGGQRDERRK
WIQCFNDVTAIIFVVASSSYNMVIREDNQTNRLQEALNLFKSIWNNRWLRTISVI

LFLNKQDLLAEKVLAGKSKIEDYFPEFARYTTPEDATPEPGEDPRVTRAKYFIRD
EFLRISTASGDGRHYCYPHFTCAVDTENIRRVFNDCRDIIQRMHLRQYELL*

GαsiN18
MGCTLSAEDKAAVERSKMIEKQLQKDKQVYRATHRLLLLGAGESGKSTIVKQ
MRILHVNGFNGDSEKATKVQDIKNNLKEAIETIVAAMSNLVPPVELANPENQFR
VDYILSVMNVPDFDFPPEFYEHAKALWEDEGVRACYERSNEYQLIDCAQYFLD
KIDVIKQADYVPSDQDLLRCRVLTSGIFETKFQVDKVNFHMFDVGGQRDERRK
WIQCFNDVTAIIFVVASSSYNMVIREDNQTNRLQEALNLFKSIWNNRWLRTISVI
LFLNKQDLLAEKVLAGKSKIEDYFPEFARYTTPEDATPEPGEDPRVTRAKYFIRD
EFLRISTASGDGRHYCYPHFTCAVDTENIRRVFNDCRDIIQRMHLRQYELL*

Gα11iN18 (Construct **a** in Supplementary Figure 1a)
MGCTLSAEDKAAVERSKMIEKQLRRDKRDARRELKLLLLGTGESGKSTFIKQM
RIIHGAGYSEEDKRGFTKLVYQNIFTAMQAMIRAMETLKILYKYEQNKANALLI
REVDVEKVTTFEHQYVSAIKTLWEDPGIQECYDRRREYQLSDSAKYYLTDVDR
IATLGYLPTQQDVLRVRVPTTGIIEYPFDLENIIFRMVDVGGQRSERRKWIHCFE
NVTSIMFLVALSEYDQVLVESDNENRMEESKALFRTIITYPWFQNSSVILFLNKK
DLLEDKILYSHLVDYFPEFDGPQRDAQAAREFILKMFVDLNPDSDKIIYSHFTCA
TDTENIRFVFAAVKDTILQLNLKEYNLV*

Construct **b** in Supplementary Figure 1a
MGCTLSAEDKAAVERSKMIDRNLRRDKRDARRELKLLLLGTGESGKSTFIKQM
RIIHGAGYSEEDKRGFTKLVYQNIFTAMQAMIRAMETLKILYKYEQNKANALLI
REVDVEKVTTFEHQYVSAIKTLWEDPGIQECYDRRREYQLSDSAKYYLTDVDR
IATLGYLPTQQDVLRVRVPTTGIIEYPFDLENIIFRMVDVGGQRSERRKWIHCFE
NVTSIMFLVALSEYDQVLVESDNENRMEESKALFRTIITYPWFQNSSVILFLNKK
DLLEDKILYSHLVDYFPEFDGPQRDAQAAREFILKMFVDLNPDSDKIIYSHFTCA
TDTENIRFVFAAVKDTILQLNLKEYNLV*

Construct **c** in Supplementary Figure 1a
MGCTLSAEDKAAVERSKMIDRNLREDKRDARRELKLLLLGTGESGKSTFIKQM
RIIHGAGYSEEDKRGFTKLVYQNIFTAMQAMIRAMETLKILYKYEQNKANALLI

REVDVEKVTTFEHQYVSAIKTLWEDPGIQECYDRRREYQLSDSAKYYLTDVDR
IATLGYLPTQQDVLRVRVPTTGIIEYPFDLENIIFRMVDVGGQRSERRKWIHCFE
NVTSIMFLVALSEYDQVLVESDNENRMEESKALFRTIITYPWFQNSSVILFLNKK
DLLEDKILYSHLVDYFPEFDGPQRDAQAAREFILKMFVDLNPDSDKIIYSHFTCA
TDTENIRFVFAAVKDTILQLNLKEYNLV*

Gα11iN (Construct **d** in Supplementary Figure 1a)
MGCTLSAEDKAAVERSKMIDRNLREDGEKARRELKLLLLGTGESGKSTFIKQM
RIIHGAGYSEEDKRGFTKLVYQNIFTAMQAMIRAMETLKILYKYEQNKANALLI
REVDVEKVTTFEHQYVSAIKTLWEDPGIQECYDRRREYQLSDSAKYYLTDVDR
IATLGYLPTQQDVLRVRVPTTGIIEYPFDLENIIFRMVDVGGQRSERRKWIHCFE
NVTSIMFLVALSEYDQVLVESDNENRMEESKALFRTIITYPWFQNSSVILFLNKK
DLLEDKILYSHLVDYFPEFDGPQRDAQAAREFILKMFVDLNPDSDKIIYSHFTCA
TDTENIRFVFAAVKDTILQLNLKEYNLV*

Construct **e** in Supplementary Figure 1a
MGCTLSAEDKAAVERSKMIDRNLREDGEKAARELKLLLLGTGESGKSTFIKQM
RIIHGAGYSEEDKRGFTKLVYQNIFTAMQAMIRAMETLKILYKYEQNKANALLI
REVDVEKVTTFEHQYVSAIKTLWEDPGIQECYDRRREYQLSDSAKYYLTDVDR
IATLGYLPTQQDVLRVRVPTTGIIEYPFDLENIIFRMVDVGGQRSERRKWIHCFE
NVTSIMFLVALSEYDQVLVESDNENRMEESKALFRTIITYPWFQNSSVILFLNKK
DLLEDKILYSHLVDYFPEFDGPQRDAQAAREFILKMFVDLNPDSDKIIYSHFTCA
TDTENIRFVFAAVKDTILQLNLKEYNLV*

Construct **f** in Supplementary Figure 1a
MTLESMMACCLSAEDKAAKERNAMIEKQLRRDKRDARRELKLLLLGTGESGK
STFIKQMRIIHGAGYSEEDKRGFTKLVYQNIFTAMQAMIRAMETLKILYKYEQN
KANALLIREVDVEKVTTFEHQYVSAIKTLWEDPGIQECYDRRREYQLSDSAKY
YLTDVDRIATLGYLPTQQDVLRVRVPTTGIIEYPFDLENIIFRMVDVGGQRSERR
KWIHCFENVTSIMFLVALSEYDQVLVESDNENRMEESKALFRTIITYPWFQNSS
VILFLNKKDLLEDKILYSHLVDYFPEFDGPQRDAQAAREFILKMFVDLNPDSDKI
IYSHFTCATDTENIRFVFAAVKDTILQLNLKEYNLV*

Gα12iN18

MGCTLSAEDKAAVERSKMIDALLARERRAVRRLVKILLLGAGESGKSTFLKQM
RIIHGREFDQKALLEFRDTIFDNILKGSRVLVDARDKLGIPWQYSENEKHGMFL
MAFENKAGLPVEPATFQLYVPALSALWRDSGIREAFSRRSEFQLGESVKYFLDN
LDRIGQLNYFPSKQDILLARKATKGIVEHDFVIKKIPFKMVDVGGQRSQRQKWF
QCFDGITSILFMVSSSEYDQVLMEDRRTNRLVESMNIFETIVNNKLFFNVSIILFL
NKMDLLVEKVKTVSIKKHFPDFRGDPHRLEDVQRYLVQCFDRKRRNRSKPLFH
HFTTAIDTENVRFVFHAVKDTILQENLKDIMLQ*

Supplementary Table Primer sequences used in this study

M2R ICL3 extension primers	
	TCTTGACGATCTTGCGTGCAACGATGCTAACGGGGTCCTGGTTAGCCACC
	TGACTAAGCAACCTGCTAAGAAGAAGCCCCCTCCATCAAGGGAG
M1R ICL3 extension primers	
	GGGCTGCCAGCTCCCGTGCTCGGTTCTCTGTCTCCCGGTAGATG
	GCCTGGCGTCTCGGAGCCCTGAAGGGCTGCCAGCTCCCGTGCTCGG
	AAGCGGAAGACCTTCTCGCTGGTCAAGGAGAAGAAGGCGGC
	GGAAAGGAGCAGCTGGCCAAGCGGAAGACCTTCTCGCTGGTC
GsiN primers	
	GCTATAACTCGAGGAGCCTGCAGTCTCGAGGCATGCGGTACC
	GGCGGTGCGTGGCCGCCGCCTTCTCGCCGTCCTCACGG
	GAAGGCGGCGGCCACGCACCGCCTGCTGCTGCTGGG
	CGAGACTGCAGGCTCCTCGAGTTATAGCAGCTCGTACTGACG
GsiN18 primers	
	AAGGACAAGCAGGTCTACCGGGCCACGCACCGCCTGCTGCTGCTGGG
	CTGCAGCTGCTTCTCGATCATCTTACTCCGCTCCACCG
G11iN primers	
	GGAGTACAACCTGGTCTGACTCGAGTCTAGAGCCTGCAGTCTCG
	GGCTCTAGACTCGAGTCAGACCAGGTTGTACTCCTTGAGGTTGAGC
Construct **a**	CAGCTGCTTCTCGATCATCTTACTCCGCTCCACCGCC
	GGAGTAAGATGATCGAGAAGCAGCTGCGGCGGGAC
Construct **b**	GCTTGTCCCGCCGCAGGTTGCGGTCGATCATCTTACTCCGC
	GATCGACCGCAACCTGCGGCGGGACAAGCGCGACG
Construct **c**	CGTCGCGCTTGTCCTCACGGAGGTTGCGGTCGATC
	GCAACCTCCGTGAGGACAAGCGCGACGCCCGGCGCG
Construct **d** (G11iN18)	GCTCGCGCCGGGCCTTCTCGCCGTCCTCACGGAGG
	GGACGGCGAGAAGGCCCGGCGCGAGCTCAAGCTGC
Construct **e**	GCAGCTTGAGCTCGCGCGCCGCCTTCTCGCCGTCCTC
	CGAGAAGGCGGCGCGCGAGCTCAAGCTGCTGCTGC
Construct **f**	AAGGAGCGCAACGCCATGATCGAGAAGCAGCTGCGGCGGG
	GGCCGCCTTGTCCTCTGCGCTCAGGCAACACGCCATCATGG
G12iN primers	
	GCTGCAGTGACTCGAGTCTAGAGCCTGCAGTC
	AGCGCGTCGATCATCTTACTCCGCTCCACCGCC
	GCGGAGTAAGATGATCGACGCGCTGCTGGCCCGCG
	CTAGACTCGAGTCACTGCAGCATGATGTCCTTC
